# Rift Valley Fever Phlebovirus Reassortment Study in Sheep

**DOI:** 10.3390/v16060880

**Published:** 2024-05-30

**Authors:** Velmurugan Balaraman, Sabarish V. Indran, In Joong Kim, Jessie D. Trujillo, David A. Meekins, Vinay Shivanna, Michelle D. Zajac, Kinga Urbaniak, Igor Morozov, Sun-Young Sunwoo, Bonto Faburay, Klaus Osterrieder, Natasha N. Gaudreault, William C. Wilson, Juergen A. Richt

**Affiliations:** 1 Center of Excellence for Emerging and Zoonotic Animal Diseases, Diagnostic Medicine/Pathobiology, College of Veterinary Medicine, Kansas State University, Manhattan, KS 66506, USA; balarama@vet.k-state.edu (V.B.); sabarish.indran@gmail.com (S.V.I.); jdtrujillo@vet.k-state.edu (J.D.T.); sunwoosy@gmail.com (S.-Y.S.);; 2 Foreign Arthropod-Borne Animal Diseases Research Unit, United States Department of Agriculture, Agricultural Research Service, National Bio and Agro-Defense Facility, Manhattan, KS 66505, USA

**Keywords:** Rift Valley fever phlebovirus, bunyavirus, sheep, reassortment

## Abstract

Rift Valley fever (RVF) in ungulates and humans is caused by a mosquito-borne RVF phlebovirus (RVFV). Live attenuated vaccines are used in livestock (sheep and cattle) to control RVF in endemic regions during outbreaks. The ability of two or more different RVFV strains to reassort when co-infecting a host cell is a significant veterinary and public health concern due to the potential emergence of newly reassorted viruses, since reassortment of RVFVs has been documented in nature and in experimental infection studies. Due to the very limited information regarding the frequency and dynamics of RVFV reassortment, we evaluated the efficiency of RVFV reassortment in sheep, a natural host for this zoonotic pathogen. Co-infection experiments were performed, first in vitro in sheep-derived cells, and subsequently in vivo in sheep. Two RVFV co-infection groups were evaluated: group I consisted of co-infection with two wild-type (WT) RVFV strains, Kenya 128B-15 (Ken06) and Saudi Arabia SA01-1322 (SA01), while group II consisted of co-infection with the live attenuated virus (LAV) vaccine strain MP-12 and a WT strain, Ken06. In the in vitro experiments, the virus supernatants were collected 24 h post-infection. In the in vivo experiments, clinical signs were monitored, and blood and tissues were collected at various time points up to nine days post-challenge for analyses. Cell culture supernatants and samples from sheep were processed, and plaque-isolated viruses were genotyped to determine reassortment frequency. Our results show that RVFV reassortment is more efficient in co-infected sheep-derived cells compared to co-infected sheep. In vitro, the reassortment frequencies reached 37.9% for the group I co-infected cells and 25.4% for the group II co-infected cells. In contrast, we detected just 1.7% reassortant viruses from group I sheep co-infected with the two WT strains, while no reassortants were detected from group II sheep co-infected with the WT and LAV strains. The results indicate that RVFV reassortment occurs at a lower frequency in vivo in sheep when compared to in vitro conditions in sheep-derived cells. Further studies are needed to better understand the implications of RVFV reassortment in relation to virulence and transmission dynamics in the host and the vector. The knowledge learned from these studies on reassortment is important for understanding the dynamics of RVFV evolution.

## 1. Introduction

Rift Valley fever phlebovirus (RVFV) is a zoonotic pathogen that was first identified in the Great Rift Valley in Kenya [1,2], and later reported in more than 15 African countries [3] and Saudi Arabia [4]. RVFV causes Rift Valley fever (RVF) in a variety of animal species, including cattle, goats, sheep, camels, buffaloes, and experimentally in white-tailed deer [5,6,7,8]. Infections can result in abortions, fetal malformations, and acute lethal infections in neonates and juveniles [9,10]. Humans develop a febrile disease that can progress to a potentially fatal hemorrhagic condition and/or a neurological syndrome [11,12,13]. RVFV is mainly transmitted through RVFV-infected mosquito bites (predominantly by *Aedes* and *Culex* spp.) or by direct contact with infected animal blood and/or tissues [11,14,15]. Fifty mosquito species have been identified as potential vectors for RVFV, and 47 species have been demonstrated to be competent vectors for RVFV transmission in experimental studies [16,17].

RVFV is a negative or ambisense RNA virus belonging to the family *Phenuiviridae*, genus *Phlebovirus*, with a tri-partite genome, which consists of three segments: large (L; 6.4 kb), medium (M; 3.8 kb), and small (S; 1.7 kb). The L segment encodes for the viral RNA polymerase gene, the M segment encodes for the non-structural NSm protein and the virus envelope proteins Gc and Gn, and the S segment encodes for the nucleoprotein (N) in the sense orientation and the non-structural NSs protein in the anti-sense orientation. RVFVs have high genetic homology with minor variation at the nucleotide and amino acid level [3,18,19,20]. However, multiple genetic lineages of RVFV exist in nature [21,22] and have been shown experimentally to exhibit differences in virulence in mice [22] and in sheep [6] and calves [23].

Reassortment (RA) refers to the exchange of genetic segments among closely related viruses. This process has the potential to yield novel viruses with modified transmission, infection, and pathogenicity characteristics. In viruses with segmented genomes, such as RVFV, reassortment can occur during replication in co-infected cells. This results in the generation of genetically distinct progeny viruses that carry genomic segments from different parental virus strains [24,25]. Sequencing and phylogenetic analysis have shown the occurrence of RVFV reassortment during epidemics [18,19,26,27]. RVFV reassortment has also been demonstrated experimentally in mosquitoes [24] and in in vitro studies in mammalian-derived cell cultures and mosquito cells [28].

Reassortment plays a pivotal role in bunyavirus evolution and diversity [29,30,31]. Currently, RVF vaccines are not used in non-endemic countries, but live attenuated and inactivated vaccines are used for veterinary use in endemic countries during and between RVF outbreaks [32,33]. Three live attenuated RVF vaccines are licensed and used, or have been pursued for development: the Smithburn vaccine, Clone-13, and MP-12 [34,35]. Reassortment between a vaccine and field strains has the potential to give rise to new viral genotypes with different phenotypes, such as altered virulence and transmission potential or impacts vaccine efficacy [28]. However, it has been suggested that reassortment between an attenuated vaccine strain and a circulating virulent strain may not necessarily result in genotypes with increased virulence but rather results in lower virulent strains [36]. Therefore, reassortment should not deter the use and the development of live attenuated vaccine (LAV) RVFV vaccines. Nonetheless, there is a lack of knowledge regarding the frequency of reassortment in general, and more specifically between two virulent strains or between an attenuated vaccine strain and a virulent strain in susceptible animals.

Here, we evaluated RVFV reassortment after experimental co-infections of sheep-derived cells or sheep with two wild-type virulent strains: Kenya 128B-15 (Ken06) and Saudi Arabia SA01-1322 (SA01), or with a live attenuated vaccine strain, MP-12, and Ken06. These studies are crucial for assessing the risk of using LAV vaccines, particularly in endemic countries where multiple wild-type RVFVs and potentially unknown phleboviruses are in circulation. Additionally, these studies provide insights about the frequency of RVFV reassortment and RVFV evolution in mammals.

## 2. Materials and Methods

### 2.1. Cells and Viruses

Madin-Darby ovine kidney cells, (MDOK; ATCC^®^ CRL-1633™; American Type Culture Collection, Manassas, VA, USA), Vero MARU cells (VM; Middle America Research Unit, Corozal, Panama) and MRC-5 cells (ATCC^®^ CCL-171™, Manassas, VA, USA) were cultured in complete Dulbecco’s Modified Eagle’s Medium (DMEM; Corning, New York, NY, USA), supplemented with 10% fetal bovine serum (FBS; R&D Systems, Minneapolis, MN, USA) and 1% antibiotic-antimycotic solution (Fisher Scientific, Waltham, MA, USA) at 37 °C under a 5% CO_2_ atmosphere in a cell culture incubator. Wild-type (WT) Kenya 128B-15 (Ken06) (accession numbers: KX096938, KX096939, and KX096940) and WT Saudi Arabia SA01-1322 (SA01) (accession numbers: KX096941, KX096942, and KX096943) isolates were propagated in the *Aedes albopictus* cell line, C6/36 cells (ATCC^®^ CRL-1660™, Manassas, VA, USA), and stored at −80 °C in the BSL-3+ lab at the Biosecurity Research Institute (BRI) at Kansas State University (KSU). The live attenuated RVFV vaccine (LAV) MP-12 strain (accession numbers: DQ375404, DQ380208, and DQ380154) was propagated in MRC-5 cells and stored at −80 °C in the BSL-2 lab. All virus-containing materials (cell culture supernatants, serum, and tissue homogenates) were titrated by a standard plaque assay, as described previously [6].

### 2.2. In Vitro Co-Infection Studies in MDOK Cells

**Figure 1:**
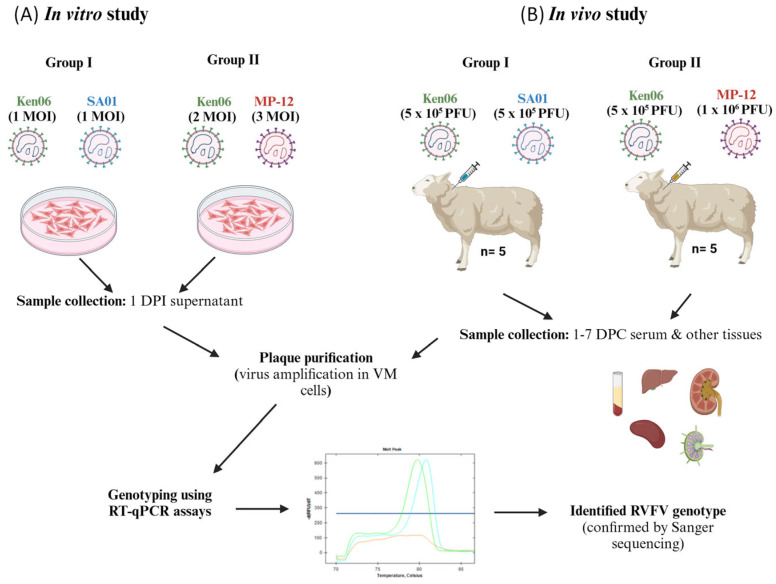
Experimental design of RVFV reassortment studies (**A**) in vitro and (**B**) in vivo. In these studies, sheep-derived cells or sheep were co-infected with two RVFV strains. The samples collected from these studies were processed, virus plaques isolated, amplified, viral RNA extracted, and segment-specific genotypes identified. WT: wild-type virulent strains Ken06 or SA01; LAV: vaccine strain MP-12; Group 1: sheep co-infected with Ken06 and SA01 strains; Group II: sheep co-infected with Ken06 and MP-12 strains; Ken06: Kenya 128B-15; SA01: SA01-1322; MOI: multiplicity of infection; PFU: plaque-forming unit; DPI: days post-infection; DPC: days post-challenge; VM: Vero MARU cells.

To study RVFV reassortment in sheep cells, we co-infected MDOK cells in a 24-well cell culture plate with the Ken06 (1 MOI) and SA01 (1 MOI) or with the Ken06 strain (2 MOI) and the MP-12 LAV strain (3 MOI), respectively (Figure 1A). The different MOIs were used to offset anticipated differences in replication kinetics between the WT and LAV strains, with the aim to provide optimal opportunity for co-infection. The viruses were allowed to adsorb for an hour at 37 °C in a cell culture incubator under a 5% CO_2_ atmosphere. Unabsorbed viruses were washed off from the cells by rinsing twice with complete DMEM, and 0.5 mL of complete DMEM was added onto the cells. Infected cell supernatants were collected at 1 day post-infection and stored at −80 °C for further use.

### 2.3. In Vivo Co-Infection Studies in Sheep

Twelve healthy short-haired sheep, aged 3–4 months, were obtained from a commercial farm in Kansas, USA. The sheep were allowed to acclimatize for nine days at the Large Animal Research Center (LARC) at KSU. Three days prior to the start of the experiment, the animals were moved into the BSL-3Ag facility in the KSU BRI. The sheep were divided into two groups: group I consisted of sheep #50, #52, #53, #54, and #55 and group II consisted of sheep #44, #46, #47, #48, and #49. Two sheep (#56 and #57) served as mock-inoculated contact controls, one per group.

The sheep were monitored daily for rectal temperatures and clinical signs. On the day of the challenge (0 days post-challenge; 0 DPC), co-infections were performed via two separate syringes for each of the virus strains. Five animals in group I were subcutaneously inoculated with 5 × 10^5^ plaque-forming units (PFU) of the Ken06 and SA01 strains, each delivered at separate sites on the right side of the neck (Figure 1B). Similarly, five animals in group II were inoculated with 5 × 10^5^ PFU of WT strain Ken06 and 1 × 10^6^ PFU of LAV strain MP-12 (Figure 1B). The two control sheep were mock-inoculated using the same volume of sterile cell culture medium. The two groups of sheep were kept in separate pens in the same room. The sheep were observed for clinical signs and temperatures until 10 DPC, and whole blood was collected for serum from 0 to 7 DPC.

At 4 DPC, two animals from each group (#50 and #52 in group I; #44 and #47 in group II) were randomly chosen to be humanely euthanized and necropsied. At 10 DPC, the remaining animals were humanely euthanized and necropsied. The liver, spleen, right kidney, and right prescapular lymph node were collected from all the animals. Tissues for histopathology were collected in formalin, while tissues for virological and molecular biological analyses were collected on ice and stored at −80 °C until further processing.

### 2.4. Tissue Homogenization

The collected tissues were homogenized using Tissue Lyser LT (Qiagen, Germantown, MD, USA) with metallic beads, as per the manufacturer’s instructions. The resulting homogenates were stored at −80 °C for future use. The homogenates were used for virus titration by a plaque assay, virus isolation by plaque purification, or for viral RNA isolation.

### 2.5. Virus Isolation by Plaque Purification

Virus isolation involved plaque purification from RVFV-positive samples using VM cells. Briefly, appropriately diluted samples were added onto VM monolayers and incubated for an hour at 37 °C. Then, the virus infection medium was replaced with 2 mL of 1.8% agarose (Seakem LE Agarose, Lonza, Walkersville, MD, USA) solution mixed with an equal volume of 2× Minimum Essential Medium (MEM, Fisher Scientific, Waltham, MA, USA), 10% FBS (R&D Systems, Minneapolis, MN, USA), and 2% antibiotic-antimycotic solution (Fisher Scientific). The infected cells were incubated at 37 °C under 5% CO_2_ for 4 days. The cells were stained with neutral red solution (Sigma-Aldrich, St. Louis, MO, USA) in 0.9% agarose overlay medium. After 4 h, plaques were picked using a 200 µL pipette tip and transferred to a sterile cryovial containing 200 µL of complete DMEM and stored at −80 °C for further use. The isolated virus plaques were amplified in VM cells in 24-well plates, incubated for 5 days, and the supernatant was collected and stored at −80 °C.

### 2.6. RNA Isolation

Viral RNA was extracted from cell culture supernatants, serum, or homogenized tissues using two methods. For cell culture supernatants and serum, viral RNA was extracted using the QIAmp Viral RNA Mini kit (Qiagen, Germantown, MD, USA) according to the manufacturer’s instructions. For other samples, viral RNA was extracted using a magnetic bead nucleic acid extraction kit (GeneReach USA, Lexington, MA, USA) on an automated Taco^TM^ mini nucleic acid extraction system (GeneReach) according to the manufacturer’s instructions. The extracted viral RNA was stored at −80 °C for further use.

### 2.7. Next Generation Sequencing (NGS)

The 2 DPC serums collected from sheep #50, #52, #53, # 54, #55, #44, #46, #47, #48, and #49 were used for RNA extraction using the QIAmp Viral RNA Mini kit (Qiagen, Germantown, MD, USA) following the manufacturer’s protocol. Briefly, 5 µL of extracted RNA was used for one-step RT-PCR amplification using the one-step RT-PCR superscript III kit (Fisher Scientific, Waltham, MA, USA) with the following primers: L forward (5′ GTTATCCGTGACAATTTCTCCCG 3′), L reverse (5′ CATCTCCACCTCTTCCTTTCTCAG 3′), M forward (5′ TCAGAAACAGACCAGGGAAGGG 3′), and M reverse (5′ AGCTCCCTCTTGGTCTGACC 3′) for the L and M segments, respectively. The amplified products were 681 bp and 724 bp in size for the L and M segments, respectively, and were purified by using the QIAquick PCR purification kit (Qiagen, Germantown, MD, USA). The concentration of the PCR products was measured using a spectrophotometer (Fisher Scientific, Waltham, MA, USA). The L and M segment amplicons of each sample were mixed in equal proportion based on their copy numbers. The products were subjected to sequencing library prep using Nextera XT (Illumina) and sequenced by next generation sequencing (NGS) using the Illumina MiSeq with 150 bp paired-end reads.

### 2.8. Quantification and Genotyping of RVFV

The RVFV RNA genome was quantified using an RT-qPCR assay based on a previously published procedure [36]. The qScript XLT One-Step RT-PCR (2×) master mix (Quanta Biosciences, Beverly, MA, USA) was used along with RVFV genomic RNA-specific primers [35]. Genotyping assays based on melt curve analysis were performed as previously described for the identification of RVFV parental strains and their reassortant viruses [37]. The genotyping assays utilize a one-step RT-qPCR mix with strain-specific primers and a common primer for each of the three RVFV genomic segments. These primers have long or short G/C tags to produce amplicons with distinguishable melt curves. After PCR, amplicons with unique melting temperature signatures are generated, allowing for strain identification via post-PCR melt curve analysis. Briefly, the genotyping primers were mixed with 10 µL of qScript XLT 2× mix, 1 µL of Eva green (20×) dye (VWR, Radnor, PA, USA), 2.5 µL of RNA template, and nuclease-free water for a total volume of 20 µL. The amplification and determination of the melting curve were carried out using the CFX thermocycler (Bio-Rad, Hercules, CA, USA). The melting curves were analyzed using CFX 3.1 software (Bio-Rad, USA). Based on the peak melting temperature, the samples were categorized as WT strains Ken06 (79.2 °C/81.8 °C/79.8 °C) or SA01 (75.4 °C/79.8 °C/80.8 °C) from group I co-infection or the LAV MP-12 (80.4 °C/75.8 °C/81.4 °C) or Ken06 (77 °C/79 °C/78.2 °C) strains from group II co-infection for the L or M or S segment, respectively. All the identified reassortant plaques were confirmed by Sanger sequencing, as previously described [37].

## 3. Results

### 3.1. RVFV Reassortment in MDOK Sheep Cells

**Figure 2:**
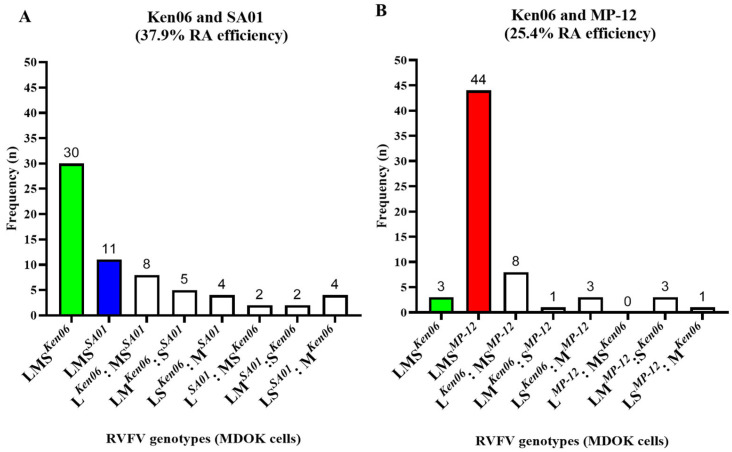
In vitro RVFV co-infections in MDOK sheep cells. The MDOK sheep kidney-derived cell line was co-infected with either the (**A**) two wild-type (WT) strains (Ken06; SA01), or (**B**) one WT (Ken06) and one LAV vaccine (MP-12) strain. Plaque-isolated viruses from the cell culture supernatants were genotyped. n: number of plaque genotypes identified; LMS: large, medium, and small segment of RVFV; Ken06: WT strain, Kenya 128B-15; SA01: WT strain, Saudi Arabia SA01-1322; MP-12: vaccine strain; RA: reassortment; MDOK: Madin-Darby ovine kidney cells.

The efficiency of RVFV reassortment was evaluated in vitro by RVFV co-infection experiments in sheep-derived cells. Specifically, co-infections with different RVFV strains in MDOK cells consisted of two wild-type (WT) strains, Ken06 and SA01, in group I, and with Ken06 and the LAV vaccine strain MP-12 in group II (Figure 1A). Supernatants from infected cells were collected 24 h post-infection and virus titers were determined by a plaque assay. The titers were 5 × 10^3^ PFU/mL for the group I co-infections and 1.75 × 10^4^ PFU/mL for the group II co-infections. Subsequently, individual viruses were isolated from the supernatants via plaque purification in VM cells. Cumulatively, 69 and 66 plaques were isolated from the group I and group II co-infections, respectively. The isolated virus plaques were expanded, and RNA was extracted and genotyped. The genotypes of three plaques from both groups had melt curves representing both the parental strains, indicative of non-pure plaque isolates, and were therefore excluded from further analysis. The results showed that 37.9% of the plaques were reassortant viruses (RAVs) from the group I co-infection and 25.4% were RAVs from the group II co-infection. Specifically, 25 plaques were RAVs, 30 plaques were parental Ken06 isolates, and 11 plaques were parental SA01 isolates from the group I co-infection (Figure 2A). From the group II co-infection, 16 plaques were RAVs, 44 plaques were parental MP-12 isolates, and 3 plaques were parental Ken06 isolates (Figure 2B). The genotypes obtained of all RAVs were further confirmed by Sanger sequencing. In summary, our results suggest that all three RVFV strains, Ken06, MP-12, and SA01, have the inherent capability to generate reassortant viruses in MDOK sheep cells upon co-infection.

### 3.2. RVFV Reassortment in Sheep In Vivo

Reassortment efficiency was evaluated in vivo using an established RVF sheep model [5,38]. Briefly, five sheep (#50, #52, #53, #54, and #55) were co-infected with the group I WT strains, and another five sheep (#44, #46, #47, #48 and #49) were co-infected with the WT and the LAV group II strains (Figure 1B).

All sheep in both groups I and II developed fever and viremia from 2 DPC onwards (Figure S1). Typical RVFV-induced macro- and microscopic lesions were observed in sheep from both groups, as in previous studies with this model [5,6], and no significant difference was noted between the two groups (File S1).

To confirm and monitor RVFV infection, serum samples were first tested from all sheep for the presence of viral RNA via the RT-qPCR assay. RVFV-specific RNA was detected in all group I and group II sheep from 1 DPC until 4 DPC, and up to 7 DPC in some of the remaining sheep (#46, #53 and #54; (Table 1)). A 1000- to 10,000-fold increase in virus copy numbers was observed at 2 DPC when compared to 1 DPC sera from all sheep in group I and group II. Following the peak RNAemia on 2 or 3 DPC, viral copy numbers started declining from 3 DPC onwards in all sheep. After 4 DPC, viral RNA was detected in the majority (4/6) of the remaining sheep (sheep #46, #49, #53, and #54), independent of the group (Table 1).

Plaque assays to determine infectious virus titers were also performed on serum and tissue sample homogenates for all group I and group II sheep (Table 2). The majority of sheep sera were positive already by 1 DPC, and all the group I and II sheep had infectious virus detected in serum by 2 and 3 DPC (Table 2). The titers corresponded well with the viral RNA copy numbers (Table 1). Conversely, only a few sheep (#50, #52, #44, and #47) had infectious virus in tissues collected on 4 DPC (Table 2). Overall, the data indicate that all the group I and group II sheep were productively infected with RVFV.

Next-generation sequencing (NGS) was used to sequence the RVFV L and M segments of the parental RVFV strains in the 2 DPC serum samples from all sheep of both co-infection groups. The results revealed that in group I, two of the five sheep (#50 and #55) had an average of 99.5% of reads mapping to Ken06 and an average of 0.5% of reads mapping to the SA01 genome. Interestingly, the other three sheep (#52, #53, and #54) had 86.3–96.5% of reads specific to Ken06, while 3.5–13.7% of reads mapped to SA01 (Table 3). All the group II sheep had an average of 99.97% of reads mapping to the Ken06 genome and an average of 0.03% of reads mapping to the MP-12 genome (Table 3). Overall, the results indicate that both groups of sheep were productively infected with RVFV, and in both groups the Ken06 wild-type virus was the predominant virus on 2 DPC.

To identify RAVs, plaque isolation and established genotyping assays [37] were performed on serum and tissue samples collected from the co-infected sheep (Table 4).

**Figure 3:**
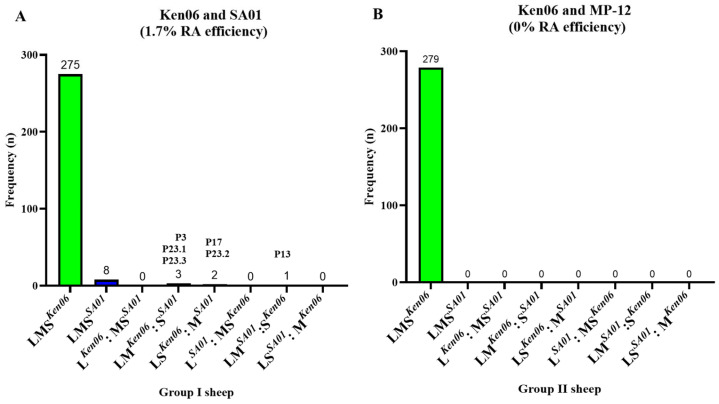
RVFV co-infection in sheep. Group I and II sheep were co-infected with either (**A**) two wild-type (WT) strains, or (**B**) a WT and LAV strain. Plaque-isolated viruses from the tissues were genotyped. n: number of plaque genotypes identified; LMS: large, medium, and small segments of RVFV; Ken06: WT strain, Kenya 128B-15; SA01: WT strain, Saudi Arabia SA01-1322; MP-12: vaccine strain; RA: reassortment; P3, P13, P17, P23.1, P23.2, and P23.3: isolated plaque numbers.

A total of 288 plaques were isolated from the samples of group I sheep #50 (n = 36), #52 (n = 144), #53 (n = 36), #54 (n = 36), and #55 (n = 36). Among these, 275 plaques were genotyped as Ken06, eight as SA01, and five as RAVs. Notably, only two out of the five sheep (#52 and #53) showed the presence of RAVs, as indicated in Table 4 and Figure 3A. Specifically, three plaques (P3, P13, and P23) from sheep #52 and one plaque (P17) from #53 were genotyped as RAVs (Figure 3A). Moreover, one plaque (P23) from sheep #52 exhibited a mixed melting curve of the L segment, and a second round of plaque purification was carried out, resulting in the isolation of three plaques (P23.1, P23.2, and P23.3). Among these, plaques P23.1 and P23.3 shared the same RAV genotype of LS*^Ken06^* and M*^SA01^*, while plaque P23.2 had the RAV genotype of LM*^Ken06^* and S*^SA01^*. All RAV genotypes were further confirmed through Sanger sequencing.

In total, 95% of the plaques isolated from group I sheep were genotyped as Ken06, 3% were genotyped as SA01, and 1.7% were genotyped as RAVs. This result strongly correlates with the NGS sequencing results (Table 3), where only sheep #52 and #53 had a higher representation (> 6%) of the SA01 strain in their serum as compared to the other group I sheep (Table 3).

A total of 279 plaques from the group II sheep #44 (n = 96), #46 (n = 19), and #47 (n = 164) were isolated. All were genotyped as the parental Ken06 strain, while no MP-12 or RAVs were detected (Table 4; Figure 3B). This data correlated with NGS deep sequencing data (Table 3), where only the presence of WT strain Ken06 was confirmed in the serum on 2 DPC. Based on the sequencing results, plaque purification and further analyses of sheep #48 and #49 were not warranted (Table 3).

Overall, the results indicate that co-infection of sheep with two WT RVFV strains can lead to the emergence of RAVs. However, no RAVs were detected from the sheep that were co-infected with the MP-12 vaccine strain and the WT Ken06 strain.

## 4. Discussion

RVFV reassortment has been reported and shown to occur both in nature [6,18,19,26,27] and in vitro [24,28]. However, there is limited knowledge about the dynamics of reassortment in susceptible animal species. Specifically, evaluating the risk of using live attenuated vaccines frequently used in endemic areas, where virulent RVFV strains are concurrently in circulation, is of relevance. Therefore, in this study, we aimed to understand the efficiency of reassortment between different RVFV strains in a sheep kidney-derived MDOK cell line and in sheep, a natural host for the virus.

We first evaluated the reassortment efficiency of different RVFV isolates (Ken06, MP-12, and SA01) in a cell culture. RAVs were isolated from MDOK cells co-infected with the two WT strains, Ken06 and SA01 (group I), as well as cells co-infected with the WT Ken06 strain and the MP-12 vaccine strain (group II). There were 12.5% more RAVs isolated after co-infection with the two WT strains compared to the cells co-infected with the Ken06 and MP-12 strains. Interestingly, the predominant RAV genotypes identified from MDOK cells were L*^Ken06^*: MS*^SA01^*, or L*^Ken06^*: MS*^MP^*^-*12*^, which accounted for 32% or 50% of the total RAV genotypes from group I and group II co-infections, respectively (Figure 2). These results may suggest that there is preference for the L segment selection from Ken06 and the M and S segments from the other co-infecting virus. In line with our observation, Ly et. al. (2017) have also noted the higher percentage (28%) of RAV genotypes with L segment selection from one parental RVFV strain, while the other segments were selected from the respective co-infecting strain [28]. However, the underlying reason for this preference is not clear at present; future studies are needed to determine the basis of this selection.

In comparison, the percentage of RVFV RAVs generated in ovine MDOK cells (37.9% and 25%) was lower than that in mosquito cells (83%), as has been reported previously [28]. This difference in reassortment efficiency could be attributed to either the specific virus strains employed, or the varying susceptibility of mammalian cells (ovine MDOK) compared to mosquito-derived C6/36 cells. Notably, *Culex* mosquitoes and *Aedes albopictus*-derived C6/36 cells are known to readily form RVFV reassortant viruses during co-infection [24,25,28]. Despite this, our study demonstrates that the virus strains utilized are indeed capable of reassortment in mammalian cells derived from sheep kidney.

Next, we evaluated the efficiency of RVFV reassortment in sheep. The group I and group II sheep were co-infected with two WT RVFV strains and a WT and LAV vaccine strain, respectively; they all developed typical RVFV pathology, and no pathological differences were observed between the group I and group II sheep. The RA efficiency for co-infection with the two WT strains was 1.7% (five RAVs/288 virus plaques genotyped), compared to 37.9% (25 RAVs/66 virus plaques genotyped) in co-infected MDOK sheep cell cultures. The preference observed with respect to the reassortant genotype from co-infected sheep trended towards M or S segment selection from SA01 and the other segments (L and S or L and M) from the Ken06 strain. Only in two out of the five co-infected sheep (#52 and #53) from group I RAVs genotypes were detected, and the proportion of the parental Ken06 and SA01 viruses determined by NGS at peak viremia in these animals were 86–93% and 7–14%, respectively.

No RAVs were detected in 279 plaques analyzed from the group II sheep. In contrast, the RA efficiency in co-infected MDOK cells was 25.4% (16 RAVs/63). Importantly, only a negligible percentage of MP-12 was detected by NGS in the serum of these animals compared to the WT Ken06 strain. This is consistent with previous studies that have shown that MP-12 vaccination did not produce detectable viremia in sheep or calves [39,40]. The absence of RAVs in group II sheep could be due to the difference in the replication kinetics of these two RVFV strains in sheep versus sheep cell cultures. Additionally, it is likely that Ken06 outcompeted MP-12 in infected sheep, thereby decreasing the possibility of co-infection and reassortment.

Overall, these results indicate that the potential for reassortment between WT RVFV strains in mammals is certainly possible. In contrast, reassortment between a vaccine strain, MP-12, and a WT strain, Ken06, in sheep is not very likely. However, the risk of reassortment between other live attenuated vaccine formulations (e.g., Smithburn LAV vaccine) and WT RVFV strains should still be evaluated as differences in dose and/or replication kinetics could influence reassortment frequency. Future studies will focus on the efficiency of reassortment in biological vectors (mosquitoes) as well as the characterization of the resulting RAVs, including replication kinetics, transmission, and pathogenicity studies.

## Figures and Tables

**Table 1:** RVFV copy numbers in sera samples of group I or group II co-infected sheep and mock-inoculated controls.

Group/Co-Infected Viruses	Sheep No.:	Serum						
		1 DPC	2 DPC	3 DPC	4 DPC	5 DPC	6 DPC	7 DPC
Group I: Ken06 and SA01	50	4.6 × 10^5^	4.3 × 10^7^	7.1 × 10^4^	2.9 × 10^4^	N/A	N/A	N/A
	52	5.7 × 10^4^	2.8 × 10^7^	1.6 × 10^9^	2.2 × 10^7^	N/A	N/A	N/A
	53	4.1 × 10^6^	1.6 × 10^9^	4.7 × 10^8^	7.7 × 10^6^	5.7 × 10^5^	3.2 × 10^4^	1.1 × 10^3^
	54	1.5 × 10^5^	5.8 × 10^7^	3.5 × 10^6^	1.4 × 10^4^	1.7 × 10^2^	1.8 × 10^5^	6.8 × 10^1^
	55	8.5 × 10^3^	3.3 × 10^7^	2.9 × 10^5^	1.7 × 10^4^	ND	ND	ND
Group II: Ken06 and MP-12	44	2.0 × 10^6^	4.8 × 10^9^	3.1 × 10^7^	7.2 × 10^7^	N/A	N/A	N/A
	46	1.2 × 10^5^	2.9 × 10^8^	2.00 × 10^8^	1.4 × 10^6^	4.1 × 10^5^	2.2 × 10^3^	3.5 × 10^2^
	47	4.2 × 10^5^	2.0 × 10^9^	2.00 × 10^10^	1.7 × 10^8^	N/A	N/A	N/A
	48	4.4 × 10^3^	1.9 × 10^6^	3.9 × 10^5^	1.2 × 10^4^	ND	ND	ND
	49	1.7 × 10^5^	1.2 × 10^8^	4.9 × 10^7^	1.8 × 10^5^	7.8 × 10^2^	8.8 × 10^2^	ND
Mock-inoculated controls	56	ND	ND	ND	ND	ND	ND	ND
	57	ND	ND	ND	ND	ND	ND	ND

DPC = days post-challenge; ND = not detected or the Cq was above 39; N/A = not applicable.

**Table 2:** Virus titers in serum and tissue samples of group I and group II sheep and mock-inoculated control sheep.

Co-Infected Viruses	Sheep No.:	Serum (PFU/mL)					Tissues (PFU/mL)			
		1 DPC	2 DPC	3 DPC	4 DPC	5,6,7,10 DPC	Liver	Kidney	PSLN	Spleen
Group I: Ken06 and SA01	50 ^‡^	1.8 × 10^3^	8 × 10^4^	ND	ND	N/A	2 × 10^1^	ND	ND	6 × 10^1^
	52 ^‡^	4.0 × 10^1^	8.8 × 10^4^	4.8 × 10^5^	1.8 × 10^4^	N/A	8.6 × 10^4^	2 × 10^4^	8 × 10^1^	7.2 × 10^4^
	53	7 × 10^4^	1.6 × 10^6^	2 × 10^5^	1.6 × 10^3^	ND	ND	ND	ND	ND
	54	4.8 × 10^3^	4.2 × 10^5^	8.6 × 10^3^	ND	ND	ND	ND	ND	ND
	55	ND	8 × 10^4^	1.8 × 10^2^	ND	ND	ND	ND	ND	ND
Group II: Ken06 and MP-12	44 ^‡^	1.4 × 10^3^	1.8 × 10^6^	5 × 10^5^	ND	N/A	5.6 × 10^3^	1.0 ×10^1^	4.0 × 10^3^	7.8 × 10^1^
	46	1.6× 10^2^	3 × 10^5^	3.4 × 10^5^	ND	N/A	ND	ND	ND	ND
	47 ^‡^	1.8 × 10^3^	2.8 ×10^6^	2.2 × 10^4^	2.2 × 10^5^	ND	2.2× 10^5^	1.4 × 10^2^	ND	4.2 × 10^5^
	48	ND	5 × 10^5^	4.0 × 10^1^	ND	ND	ND	ND	ND	ND
	49	2.2 × 10^2^	1.6 × 10^6^	1.5 × 10^5^	ND	ND	ND	ND	ND	ND
Mock-inoculated controls	56	ND	ND	ND	ND	ND	ND	ND	ND	ND
	57	ND	ND	ND	ND	ND	ND	ND	ND	ND

PFU = plaque-forming units; N/A = not applicable; ND = not detected; PSLN = pre-scapular lymph node; ^‡^ necropsied on 4 days post-challenge (DPC) and rest of sheep on 10 DPC.

**Table 3:** Summary of next-generation sequencing results for RVFV RNAs in sera from both groups of sheep. The total number of reads mapped to each of the RVFV genomic segments, and the percentage of reads mapped to the L and M segments of each strain of RVFV, are shown.

Co-Infected Viruses	Sheep No.:	Total Number of Reads	Ken06 (%)	MP-12 (%)	SA01 (%)
Group I: Ken06 and SA01	50	1,244,155	99.88	N/A	0.12
	52	1,408,214	86.34	N/A	13.66
	53	1,595,115	93.25	N/A	6.75
	54	1,459,093	96.48	N/A	3.52
	55	1,665,274	99.20	N/A	0.80
Group II: Ken06 and MP-12	44	542,700	99.97	0.03	N/A
	46	547,200	99.96	0.04	N/A
	47	439,475	99.97	0.03	N/A
	48	623,932	99.97	0.03	N/A
	49	625,216	99.97	0.03	N/A

N/A = not applicable.

**Table 4:** Summary of results for the RT-qPCR genotyping assay and Sanger sequencing of plaques isolated from infected sheep tissues and serum samples. The RAV-positive number of plaques/total number of plaques genotyped are indicated.

Co-Infected Viruses	Sheep No.:	Liver	Spleen	Serum 2 DPC	Kidney	PSLN
Group I: Ken06 and SA01	50	N/A	N/A	0/36	N/A	N/A
	52	0/36	0/36	3/36	1/36	N/A
	53	N/A	N/A	1/36	N/A	N/A
	54	N/A	N/A	0/36	N/A	N/A
	55	N/A	N/A	0/36	N/A	N/A
Group II: Ken06 and MP-12	44	0/24 ^§^	0/24^§^	0/24 ^§^	N/A	0/24 ^§^
	46	N/A	N/A	0/19 ^§^	N/A	N/A
	47	0/24 ^§^	0/24^§^	0/92	0/24 ^§^	N/A
	48	N/A	N/A	NP	N/A	N/A
	49	N/A	N/A	NP	N/A	N/A

N/A = not applicable, as these samples were negative for virus on plaque assay; NP = not performed; PSLN = pre-scapular lymph node; ^§^ = plaque purified twice, and the rest of the samples were plaque purified once.

## Data Availability

The original contributions presented in the study are included in the article/supplementary material, further inquiries can be directed to the corresponding authors.

## Supplementary Materials

The following supporting information can be downloaded at: https://www.mdpi.com/article/10.3390/v16060880/s1. File S1: Gross pathology and histopathological lesions in group I and group II sheep; Figure S1: Body temperature of mock-inoculated controls, group I (Ken06 and SA01), and group II (Ken06 and MP-12) co-infected sheep. Figure S2. Typical RVFV histopathological changes in the liver at 4DPC include multifocal to coalescing, centrilobular to midzonal hepatocellular necrosis.
