# Supplementary material for: Rift Valley Fever Phlebovirus Reassortment Study in Sheep
Source: Viruses. 2024 May 30;16(6):880. doi: 10.3390/v16060880 (PMC11209395; doi:10.3390/v16060880)
Supplement: Supplementary file 1 [file viruses-16-00880-s001.zip › viruses-2957787-supplementary.pdf]

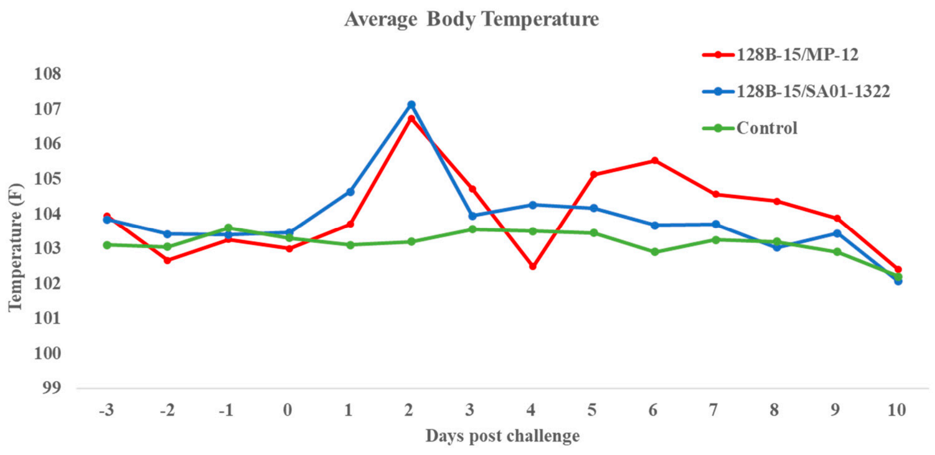

Figure S1: Body temperature of mock-inoculated controls, group I (Ken06 and SA01) and group II (Ken06 and MP-12) co-infected sheep. Daily rectal body temperatures were recorded for all sheep during the study. 128B-15: Ken06 strain; MP-12: Vaccine strain; SA01-1322: SA01 strain, and control- mock-inoculated control sheep.

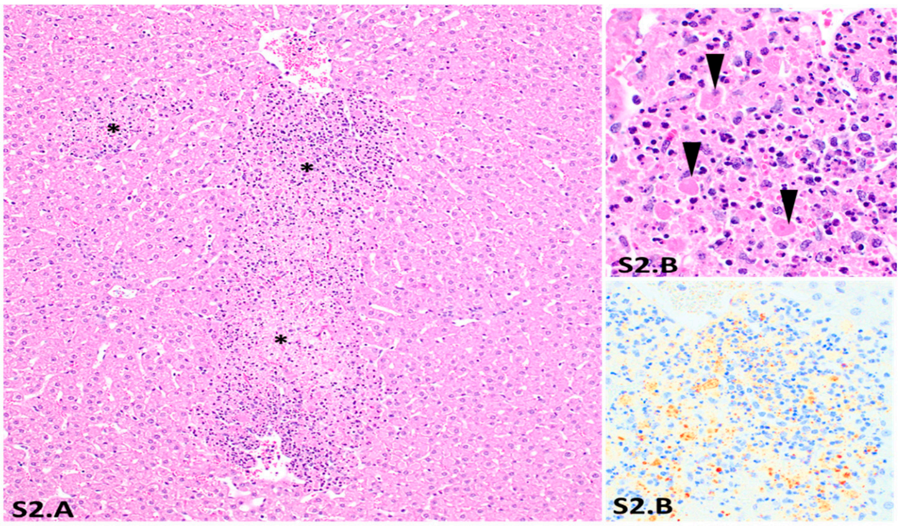

Figure S2. Typical RVFV histopathological changes in the liver at 4DPC include multifocal to coalescing, centrilobular to midzonal hepatocellular necrosis (asterisks). Foci are characterized by loss of tissue architecture and disruption of hepatic cords and consist of aggregates of degenerate cellular debris, hyper-eosinophilic, swollen individual degenerate hepatocytes (arrowheads), Mixed inflammatory cells and occasionally hemorrhage (Hematoxylin and Eosin: S2.A-100X and S2.B-200X. Immunohistochemistry straining using Anti-RVFV polyclonal rabbit serum demonstrate antigen positive staining of degenerate cellular debris in necrotic foci (Hematoxylin counter stain: S2C-100X).

File S1: Description of macroscopic and microscopic lesions in group I and group II Sheep.

(i) Macroscopic pathological observations

Two contact control animals remained clinically within normal limits with no significant macroscopic observations. In the RVFV-challenged sheep, macroscopic lesions characterized by

necrosis and hemorrhage in the liver and pre-scapular lymph nodes were severe at 4 DPC and completely resolved at 10 DPC in both Groups I and II. Notably, Group II animals exhibited higher severities at 4 DPC, possibly correlated with pronounced clinical signs compared to Group I animals.

At 4 DPC, two animals from each group were euthanized and necropsied. The liver, pre-scapular lymph node, and spleen were the most severely affected organs in both Group I and Group II. Commonly observed lesions included multifocally scattered 1-2 mm petechiae and necrosis throughout the hepatic parenchyma (2/2), diffuse edema and medullary hemorrhage in the right prescapular lymph node (2/2), and capsular hemorrhage in the spleen (2/2). Notably, Animal #47 in Group II exhibited most severe macroscopic lesions, including multifocal areas of edema (200-300 mL peritoneal effusion, edema in the mesocolon, mesentery, gallbladder, and pericardium), as well as hemorrhages in the lymph nodes, liver, splenic capsule, and gastrointestinal serosa with higher severity in the rumen and abomasum.

At 10 DPC, two animals (#48, #49) in Group II had non-specific hepatic lesions. Animal #48 had a locally extensive area (4 cm x 3 cm) with multiple granulomas surrounded by a fibrous capsule and yellow tan caseous exudate in the right lobe of the liver, consistent with granulomas. Animal #49 had focal fibrosis on the hepatic capsule, likely attributed to previous parasitic migration. These lesions were considered non-RVFV related. In Group I, Animal #53 had 150-200 peritoneal effusion likely attributed to resolved hepatic injury by RVFV. Otherwise, no significant macroscopic lesions were observed in either group.

In controls, Animal #57 had multifocal dark red areas on the liver capsule. Upon cut sections, no significant macroscopic observation was noted in the hepatic parenchyma. The spleen was diffusely enlarged and congested and likely attributed to barbiturate euthanasia.

(ii) Histopathology

In the two contact control animals, no significant microscopic lesions were noted. Among the virus-challenged sheep, microscopic lesions included necrosis and hemorrhage centered in the liver, gall bladder, and pre-scapular lymph nodes as macroscopic observations. The lesions were severe at 4 DPC but significantly resolved at 10 DPC in both Groups I and II. Notably, Group II animals exhibited higher severities at 4 DPC, consistent with their pronounced clinical signs and macroscopic findings at 4 DPC.

At 4 DPC, animals in both groups I and II displayed the most severe lesions in the liver, pre-scapular lymph node, and spleen (44, 50;).

In the liver, affected areas were approximately 35% and 10% of the examined section in the group II and group I, respectively. These areas were characterized by multifocal, random areas of lytic necrosis with low to moderate numbers of viable neutrophils, cellular debris, and hemorrhage. In the periphery in within regions of the necrosis, hepatic cords were dissociated, and hepatocytes were with hypereosinophilic, rounded and individualized (degeneration) with pyknosis, karyolysis, and karyorrhexis (necrosis) See SF2.

In the pre-scapular lymph node, affected areas were approximately 30-40% and 5-15% of the examined sections in group I and group II, respectively. The affected areas were characterized by multifocal to coalescing areas of hemorrhage, lymphoid depletion, and lymphoid cell necrosis (2/2).

At 10 DPC, the microscopic lesions observed at 4 DPC were significantly resolved except Animal #48 in Group II. Animal #48 exhibited similar severities of microscopic lesions as seen in those at 4 DPC. Additionally, non-specific lesions were noted in this animal including

multifocal to coalescing areas of granulomas characterized by a central core of necrotic cell debris and mineralization surrounded by epithelioid macrophages, multinucleated giant cells, and an outer band of lymphocytes and plasma cells. On the periphery of granulomas, mild to moderate bile duct hyperplasia and fibrosis with periportal low to moderate numbers of lymphocytes and plasma cells, viable neutrophils, and eosinophils were noted. The common bile ducts were surrounded by large numbers of eosinophils, moderate numbers of lymphocytes and plasma cells.
